# How Knowledge Stock Exchanges can increase student success in Massive Open Online Courses

**DOI:** 10.1371/journal.pone.0223064

**Published:** 2019-09-26

**Authors:** Andreas Heusler, Dominik Molitor, Martin Spann

**Affiliations:** 1 Ludwig-Maximilians-University of Munich, Institute of Electronic Commerce and Digital Markets, Munich, Germany; 2 Gabelli School of Business, Fordham University, New York, NY, United States of America; Universitat de Valencia, SPAIN

## Abstract

Massive Open Online Courses (MOOCs) allow lecturers to overcome spatiotemporal boundaries and reach large numbers of participants. However, the completion rates of MOOCs are relatively low, a critical obstacle to their ultimate success. Existing literature suggests that strengthening student interaction has the potential to increase student commitment. The goal of this study is to develop a novel, market-based knowledge-sharing method that fosters student engagement and interaction in MOOCs, addressing the problem of low completion rates and demonstrating how MOOC engagement can lead to greater student success. The proposed method, “Knowledge Stock Exchange” (KSX), is derived from the concept of crowd-based intelligence mechanisms for incentive-compatible information aggregation. Using a popular MOOC as the focus of our empirical study, we show that the KSX method increases student interaction as well as MOOC completion rates. Moreover, we find that KSX participation has a significant positive effect on participants’ exam grades.

## Introduction

Massive Open Online Courses (MOOCs) received considerable attention in higher education when companies such as edX, Udacity and Coursera began offering courses that attracted tens of thousands of students [1, 2]. Enabled by digital technologies, MOOCs provide a new level of scalability and open access to the course materials of top universities across the globe [2]. In terms of potential, MOOCs can be seen as an instrument for bridging the gap of temporal, geographical and social distances between students, lecturers and institutions.

Although many MOOCs exceed a participant level of 10,000 students, most of these same MOOCs show only modest completion rates of below 10 percent [3]. Initially, MOOCs were propagated in combination with the pedagogical approach of connectivism which demands a high level of social connectedness and interactivity [4, 5]. However, the next generation of MOOCs has focused on the demand for scalability, establishing such practices as video lectures, computer-graded exams and quizzes (with low collaboration among students) [2]. Recent studies hence attribute the low completion rates to the low student interaction [6, 7].

Beyond MOOCs, the question of how to motivate people to collaborate and share their private knowledge is an important topic in different fields of research [8, 9–11]. The threat of participants’ free-riding behavior and the design of targeted incentive mechanisms for knowledge sharing on platforms are key topics in e-learning and knowledge management [11]. To address these challenges, past research has experimented with the application of game design elements in organizations [12] as well as communities [13]. The use of digital game design elements in organizational contexts–also known as gamification–offers the potential to increase motivation and engagement in two dimensions: by enabling (1) more competitive and (2) more cooperative interactions [14]. Therefore, an incentive-compatible knowledge sharing method, which combines cooperative and competitive components (i.e., coopetitive), may offer a learning environment that stimulates engagement [15] and social interaction among course participants, eventually leading to greater commitment and better learning results [16–19].

In this article, we develop and evaluate an incentive-compatible knowledge sharing method–the Knowledge Stock Exchange (KSX)–that aims at increasing student completion rates in the context of MOOCs by combining cooperative (e.g., via forums) and competitive (e.g., via leaderboards) components. More specifically, the KSX is supposed to enable a more engaging and interactive learning experience by providing the opportunity to propose, trade, and discuss “virtual knowledge stocks,” in order to improve completion rates as well as exam grades. We conduct a field study that combines a large MOOC with the proposed KSX method to evaluate the method’s effectiveness. *The results of the field study answer two substantial research questions*: *Does the KSX help to improve the MOOC completion rate*? *And does the KSX affect the MOOC’s final exam grades*? We contribute to the field of e-learning and knowledge management by combining a scalable knowledge sharing method, the KSX, with a MOOC to incentivize student engagement and interaction, thereby addressing the problem of low completion rates and course success.

## Theory and prior research

In the following, we focus on previous research on massive open online courses (MOOCs) in the context of completion rates. We then introduce the concept of virtual stock markets (VSM), which is a crowd-based intelligence mechanism that is well suited to increase engagement and completion rates in MOOCs. Finally, our research model combines the gamification-based coopetitive elements of crowd-based intelligence mechanisms, student engagement and interactions as well as student success.

### Massive open online courses

Starting in 2008, the “Connectivism and Connective Knowledge” course (CCK08) was among the first to include the constituting elements of a MOOC [20]. While “massive” stands for their degree of scalability, the notion of “open” leaves room for interpretation [21]. Typically, a MOOC does not charge any tuition fees, requires no formal accreditation or pre-requisites other than internet access and the motivation to learn [2].

After its launch, the CCK08 course attracted a relatively large group of 2,200 participants worldwide and was based on an open online-learning environment [22]. Moreover, when Dave Cormier first mentioned the acronym MOOC in his educational blog, it was distinctively driven by the pedagogical ideal of connectivism [5]. Later on, this led to the term “Connectivist Massive Open Online Courses” (c-MOOC), indicating an autonomous, interactive and experimental way of developing an open knowledge base that is focused on the idea of social networking.

At the end of 2011, Stanford professors Sebastian Thrun and Peter Norvig offered their free introductory Artificial Intelligence (AI) course with 160,000 enrolled students [23]. Participants from 190 different countries were attracted by the promise to receive materials, assignments and exams similar to Stanford’s original AI course. Soon, this promise was echoed by other platforms such as Coursera, edX and Udacity (co-founded by Sebastian Thrun), also offering free high-quality content from top-universities around the globe. However, these so called “AI-Stanford like Courses” or “x-MOOCs” differ from the original c-MOOCs based on a more restrictive notion of “free access to premium but copyrighted content” [23] and less interaction among students [1].

Besides their IT-enabled scalability for tens of thousands of students [24], especially x-MOOCs struggle with low student completion rates [3, 24, 25]. Although x-MOOCs enroll about 43,000 students on average (ranging from 4,500 to 226,500 students), only about 6.5 percent of students successfully complete a course [26]. As a consequence, researchers started to invest considerable efforts in identifying the main influencing factors of low completion rates.

A comprehensive review of existing empirical studies on online course dropouts identified 69 influencing factors and assigned them to three major categories: student (i.e., academic background, relevant experiences, skills and psychological attributes), course/program (i.e., course design, institutional support and interactions) and environmental factors (i.e., work commitment and supportive environment) [27]. Additional identified factors include platform (e.g., platform usability) and provider-specific factors (e.g., university reputation, lecturer characteristics) [25, 28, 29].

Given the open access policy of most MOOCs, student and environmental factors are hard to tackle and are more or less taken for granted [27]. Platform and provider factors are rather specific and are often too detailed in order to be addressed by general methodical advice [25]. Therefore, many available counterstrategies aim at addressing course/program-specific factors, which in turn often include different elements of social interaction. For example, research on the interaction among students has shown that the use of forums is positively correlated with higher student completion rates and better final exam grades [30]. Related to the course design, there is also evidence that peer assessment increases student motivation compared to automated grading [25]. To summarize, these results suggest that specific aspects of the course design, such as social interaction and engagement, might help to increase the completion rates in the context of MOOCs.

### Crowd-based intelligence mechanisms

#### Virtual stock markets

A Virtual Stock Market (VSM) is a web-based market on which securities contracts–referred to as “virtual stocks”–are traded by a crowd of people in order to gather and aggregate widely dispersed information [31]. These virtual stocks are linked to final payoff conditions and can be interpreted as bets on future events. Due to the continuous trading of shares, a stock’s reference price reflects the continuously updated value of its underlying contract and represents the crowd’s aggregated assessment about the probability of the event to occur.

The theoretical foundation of VSMs is based on the idea that asset prices in an efficient market fully reflect all value-related information available to participants and by that aggregate all expectations on the likelihood of future payoffs [32]. Furthermore, markets may also be seen as the most efficient mechanism to aggregate asymmetrically dispersed information [33, 34].

Contrary to conventional financial stock markets, VSMs can be restricted to the usage of virtual play money [35], without losing their ability to efficiently aggregate information or weakening their incentive-inducing effect on knowledgeable traders [36]. Nevertheless, most VSM studies are based on markets with relatively short trading cycles over a couple of weeks, days or even hours [37]. Knowledge about how to maintain participants’ long-term motivation (i.e., over several months), in combination with non-monetary incentives, is scarce [38].

There are several fields of applications for VSMs that are depending on the type of virtual stocks, the incentives for participation and information revelation, the intended purpose and various market design parameters [31]. The prediction of future events including election results [39], sales forecasts [40], sports forecasts [41], or scientific research [42] are summarized under the term “prediction markets.” Other VSM applications such as the sourcing and evaluation of ideas [43], or the testing of new product concepts [37] are known as “idea markets” or “preference markets.”

#### Knowledge stock exchange

In this study, we explore a new type of VSM, hereinafter referred to as the Knowledge Stock Exchange (KSX). To better differentiate between the proposed KSX method and other established types of VSMs, we highlight four distinct characteristics: First, the KSX is set up with a different purpose, making use of an incentive-compatible learning mechanism (i.e., reaching the best outcome by acting according to one’s true preferences) to motivate a crowd of permanent participants over a longer period of time (i.e., several months) as well as to cooperatively develop and evaluate an open knowledge base [44].

Second and in contrast to the fixed contract descriptions of other VSM types, each KSX-specific virtual stock represents an editable knowledge artifact, similar to an editable wiki page. This characteristic facilitates an iterative refinement of virtual stocks over the entire trading cycle. As each modification (“edit”) of a knowledge artifact changes the stock’s value and may subsequently lead to a price reaction of the market, price changes of virtual stocks can be interpreted as a prompt feedback on the current quality of a knowledge artifact.

Third, while the static nature of stocks in preference markets leads to converging prices in the sense of reaching a consensus about the value of a given idea or concept [37], the KSX incentivizes participants to continuously update their valuation due to repeated modifications of knowledge artifacts. In order to profit from arbitrage effects, participants can invest in low-quality stocks, if they are able to improve the quality of an artifact on their own.

Fourth, besides the characteristics of a crowd-based evaluation and improvement mechanism, the KSX also includes the sourcing of stocks by the crowd, not an authority. Triggered by an open call, participants are invited to voluntarily submit their solution (i.e., “knowledge stock”) to a given problem. This characteristic, which is similar to idea markets and other crowdsourcing methods [17, 45], strongly differs from the original notion of prediction and preference markets, where stocks are proposed by an authority.

### Research model

**Fig 1:**
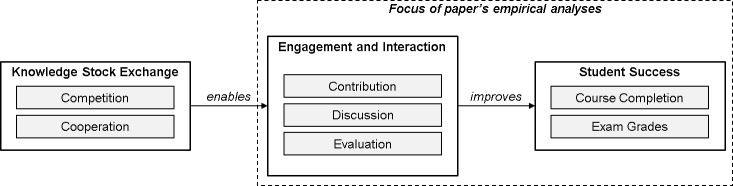
Research model.

Fig 1 shows our research model. Based on previous findings in gamification research, we make use of gamification elements including discussion forms and leaderboards, allowing us to combine cooperative and competitive components [14]. The underlying theories of competition and cooperation can be found in cognitive evaluation [46] and social comparison theory [47]. Cognitive evaluation theory states that rewards or feedback can enhance intrinsic motivation [46]. Social comparison theory in turn assumes that individuals have an innate need to evaluate their abilities in comparison with others [47].

To tab into these behaviors, we launch a Knowledge Stock Exchange as an effective and scalable mechanism to enable more student interaction on MOOCs. The interactions on the KSX platform can manifest itself by students’ active contribution to the knowledge stock (e.g., by posting tradeable solutions), forum discussions as well as peer evaluations, visualized via leaderboards. Based on previous literature [16], increased engagement and interactions–in the context of coopetive activities–are assumed to increase students’ success likelihood, measured by course completion rates and (final) exam grades. The outcomes, including measures for student engagement, interaction and student success, are the key factors of our empirical analyses.

## Method

### Knowledge stock exchange architecture

The KSX architecture consists of four main modules: (1) a communication module, providing basic forum functionalities and notification services, (2) a knowledge repository module, covering the essential functionalities of a content management system, (3) a VSM module, offering several trading and auction mechanisms, and (4) a knowledge challenges module, containing additional gamification elements. The latter includes a virtual currency (Virt$) system, a contribution point system and two corresponding leaderboards that can be used to display a performance and a contribution ranking. The integration of VSMs along with the extension of a knowledge challenges module and gamification elements is our core contribution, encouraging a coopetitive knowledge sharing and a more interactive learning experience.

### Conceptual sequence of actions in knowledge stock exchange

**Fig 2:**
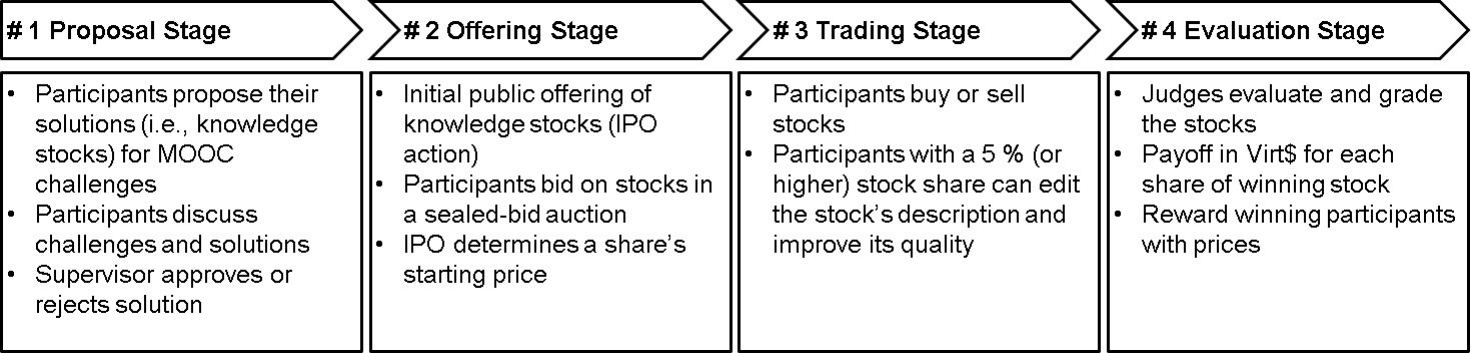
Sequence of actions in a KSX challenge.

Conceptually, the KSX consists of multiple knowledge challenges, which are independent of one another. Yet they all follow the same sequence of actions, as can be seen in Fig 2 below: (1) proposal stage, (2) offering stage, (3) trading stage, and (4) evaluation stage. In combination with a MOOC that typically contains a series of learning modules, it seems reasonable to connect each learning module with a corresponding KSX challenge. For each available learning module in the MOOC, students pass all four stages of the corresponding knowledge challenge. This allows synchronizing the KSX platform with the progress of an online course, starting a new knowledge challenge for each learning module. Moreover, due to the payoff (in virtual cash) after each knowledge challenge, students have the opportunity to get familiar with the VSM mechanism and learn about promising tactics that combine bidding, trading and editing of “[virtual knowledge] stocks.”

#### Proposal stage

In the proposal stage, a course supervisor first specifies the knowledge challenges, including a task description, a timeline, basic conditions, and evaluation criteria such as relevance to a challenge-specific problem, degree of newness, level of detail or quality of the solution. These criteria will be used by judges to determine the winning solution of a knowledge challenge (see evaluation stage). The sourcing of solutions requires the provision of a precise task description for each challenge. Typical tasks (i.e., potential solutions) comprise the creation of case studies, literature overviews, numerical examples, terminology dictionaries, as well as student exercises.

Once the knowledge challenge is posted, students are invited to propose their solutions. In doing so, they earn “contribution points,” which serve as a virtual score (see Table A in S1 File for more details). Contribution points are awarded irrespective of a solution’s quality. During the course, KSX participants can compare their current score with other participants on a leaderboard, the so-called contribution ranking. Besides proposing new solutions, participants can also upload supporting files, add ratings or comment on existing solutions which can thus be further refined and extended. Each proposed solution will be approved (or rejected) by a supervisor and converted into a so called “[virtual knowledge] stock”. Therefore, the terms “solution” and “[virtual knowledge] stock” are used interchangeably. Each challenge with its set of solutions will be treated as a separate market with a corresponding set of stocks.

#### Offering stage

All approved stocks are now offered to the participating students using a single-day sealed bid auction (i.e. “initial public offering” resp. IPO auction). This kind of IPO auction helps to determine the starting price for each stock and reduces the risk of herding behavior as all bids are hidden until the auction ends. Specifically, fixed price auction mechanisms are well-suited as they are easy to communicate and to understand by participants. Alternatively, the discriminatory or the uniform price auction could be used as IPO auctions [48].

During the offering stage, students have the option to set up their initial knowledge stock portfolios according to their personal beliefs and individual appreciation of the underlying solutions. The starting price of a stock represents the crowds’ aggregated opinion about the value of a solution and its probability to receive a final payoff in comparison to other solutions of the same challenge. As a consequence, the value of each solution is based on the predefined evaluation criteria of a challenge.

Besides the budget restrictions of their initial virtual endowments, participants do not have any further constraints on whether and how much money they invest in the offered stocks. As soon as the IPO ends, the aggregated demand of shares for each stock will be revealed in combination with the starting price.

#### Trading stage

With the start of the trading stage, all stocks are listed on the market. In order to link the prices of all stocks within the same market, the KSX uses Hanson’s logarithmic market scoring rules (LMSR) as an automated market maker algorithm [49]. As the LMSR requires a predefined maximum price (e.g., Virt$100.00), stock prices will always vary in a range between zero and the maximum price. In addition, the LMSR ensures permanent liquidity within the market [50, 51].

Depending on the number of shares circulating in the market, the LMSR calculates a reference price for each stock relative to all other stocks within the same market. This reference price indicates the current price for a minimum amount of shares. Accordingly, the stock price increases as a function of the number of ordered shares, while the reference price of all other stocks in the market decreases proportionally at the same time. Simultaneously, the reference price signals the market’s aggregated information about a stock’s probability to receive a final payoff according to the predefined evaluation criteria. Students can therefore interpret the reference price of a knowledge stock as an indicator for its current quality throughout the entire trading stage.

Similar to the contribution ranking, students can compare their financial assets in virtual currency (i.e., Virt$) via a performance ranking. Although students have to wait for the subsequent evaluation stage to receive their final payoffs, they can already realize significant arbitrage profits through the continuous trading of stocks. Starting with the initial wallet of Virt$20,000, the performance ranking keeps track of all realized and unrealized gains and losses of their trading activities.

In addition, a five percent-edit-rule allows shareholders to edit a stock’s content during the entire trading stage. A similar threshold was used in previous research [44]. This rule states that once a trader holds at least a five percent share of a stock, she is permitted to edit a stock’s description or upload supporting images and other files. Note that, as a shareholder, the trader has no incentive to decrease prizes and is rather assumed to improve the stock’s value in order to achieve arbitrage profits. Compared to conventional VSMs such as prediction, idea or preference markets that offer virtual stocks with fixed contractual specifications, the five percent-edit-rule is a key characteristic of the KSX method. It allows participants not only to trade but also to iteratively modify and improve the stocks, which are now treated as open knowledge artifacts. This extends the principle of VSMs from a crowd-based information aggregation mechanism to a crowd-based knowledge sharing mechanism. By requiring a certain degree of stock ownership, the rule also aims at increasing the costs of deliberately manipulating a shareholder’s knowledge stock.

#### Evaluation stage

In the evaluation stage, a judge (e.g., a course supervisor) assesses the quality of each stock according to the following four criteria: correctness, level of detail, comprehensibility and conciseness of a solution. These criteria are communicated in the evaluation stage.

Based on the average grade, we can determine the payoff in Virt$ for each stock. Due to our focus on the seamless communication of rules, we implement a winner-takes-all market [52]. Thereby, the solution with the best grading receives the full payoff in the respective challenge (e.g., Virt$100.00 per share). Dependent on the number of shares in their portfolio, students receive their payoffs for each winning stock.

## Field study

In order to evaluate the method’s effectiveness, we conducted a field study in collaboration with a popular MOOC on advanced management. The course was launched on Coursera, one of the largest MOOC providers, and conducted in collaboration with a partner university. The target audience of the course consisted of a broad set of learners from around the globe. Similar to other courses on Coursera, our course was promoted via e-mail newsletters to existing students, combined with search and social media marketing. The course was held over a period of two months and consisted of seven learning modules including 7.5 hours of video material. The videos largely included lectures on strategic management and strategic industry analyses. Students were required to pass a quiz for each of these modules in order to get access to the final exam and to receive the course certificate. The final exam was a multiple choice test where students could score a maximum of 30 points.

After registering for the course, students were invited to additionally register for the KSX via a forum announcement on the MOOC platform. In parallel to the entire cycle of the course, the KSX offered 12 related knowledge challenges (see Table B in S1 File) and lasted for an overall period of 70 days (from the first to the last interaction). KSX participation was voluntary. Students neither receive any kind of grade improvement nor other forms of bonuses for quizzes and/or the final exam of the course. It is worth noting that all challenges on the KSX platform were related to the actual course module(s). Thus, students were able to benefit from knowledge exchanges with their fellow students, possibly by spending additional time and effort on the KSX platform. Moreover, the Top 15 students in the leaderboards (based on contribution and performance rankings) were allowed to choose between university branded coffee mugs, backpacks and writing sets as a reward for their efforts.

### Participation, demographics and post-survey outcomes

As depicted in Table 1, the MOOC attracted a total of 61,893 registered students. From those, 992 students registered for both platforms (i.e., MOOC **∩** KSX), 60,901 students registered only for the MOOC. While most students showed some sort of activity (e.g., watching course videos or commenting in the forums), only 4,324 students (7.4%) successfully completed the course by passing seven quizzes and the final exam. Similar to participation rates of other MOOCs [26], we observed a completion rate of 6.4 percent for MOOC-only students. Students that additionally joined the KSX showed significantly higher completion rates of 64.9 percent. However, this may be driven by self-selection effects related to non-randomized groups, which we analyze in more detail below.

**Table 1:** MOOC and KSX participation.

	MOOC (Total)		MOOC only		MOOC ∩ KSX	
Registered	61,893		60,901		992	
Active Accounts	58,729	100.0%	57,808	100.0%	921	100.0%
Dropouts	54,405	92.6%	54,082	93.6%	323	35.1%
Completed	4,324	7.4%	3,726	6.4%	598	64.9%

Table 2 shows the post-survey results based on the census of participants that were registered on both the MOOC and KSX and platform (N = 364; response rate: 39.5%). On average, students stayed 207.2 minutes per week on the MOOC platform and 36.4 minutes per week on the KSX platform respectively. Based on a mean of 6.4 visits per week and 54.6 minutes per visit, participants devoted most of their time to the MOOC, compared to 2.8 visits per week and 14.6 minutes per visit on the KSX platform. Although KSX participation required a modest investment of additional time, it did not seem to harm MOOC-specific activities.

**Table 2:** Platform utilization of MOOC ∩ KSX users.

Post-Survey (N = 364)	Unit	MOOC	KSX
Time Spent	Mean (SD) Minutes per Week	207.2 (220.6)	36.4 (84.6)
Platform Visits	Mean (SD) Visits per Week	6.4 (7.3)	2.8 (7.6)
Time Spent per Visit	Mean (SD) Minutes per Visit	54.6 (75.4)	14.6 (24)

Overall, this MOOCs had a truly global audience. Students from 74 countries all over the world joined both platforms. About 83 percent of the participants were male, 17 percent female, which is similar to other business and engineering MOOCs [53]. This resulted in the following age distribution (see Fig A in S1 File): under 18 (1.1%), 18–25 (24.7%), 26–35 (37.5%), 36–45 (19.9%), 46–55 (11.2%), 56–65 (4.5%), and over 65 (1.1%). Most of the students already obtained an academic degree (see also Fig B in S1 File), which is consistent with previous research on other MOOCs [54]. Note, however, that only 7.3 percent of all surveyed participants study or already have a degree in finance and/or accounting. In addition, the mean of participants’ self-assessed stock market knowledge is 3.91 (SD = 1.62) on a scale from 1 (no knowledge) to 7 (very knowledgeable). This indicates that there does not seem to be a strong latent effect beyond self-selection with respect to the context of finance and stock markets in particular.

We also asked whether students that were registered on the MOOC and the KSX consider the KSX as “very collaborative” (M = 4.59, SD = 1.26) or “very competitive” (M = 4.57, SD = 1.26) on a scale from 1 (disagree) to 7 (agree). Both items are highly correlated (r = 0.81) which indicates that students perceive the KSX platform as coopetitive, including both collaborative and competitive elements. This is consistent with our conceptual setting.

### Platform statistics

The KSX platform offered twelve knowledge challenges (see Table B in S1 File), including seven challenges directly related to the MOOC’s seven knowledge modules. Four of the additional remaining KSX challenges referred to course-specific topics. Besides that, we also offered an introductory challenge as an opportunity to learn about the trading mechanism.

#### Forum activities

The MOOC as well as the KSX platform included forum functionalities. Table 3 shows the corresponding forum activities. On the KSX platform, 66 different commenting users started 444 forum discussions, leading to 800 comments and an average number of 12.12 comments per participant. Considering the total number of registered participants, forum interaction on the MOOC platform itself was rather low with 1,339 users posting 3.36 comments on average. By comparing between MOOC-only students (2.71 comments on average) and MOOC ∩ KSX students (6.14 MOOC comments and 12.12 KSX comments on average), we observe considerably stronger forum interactions for the latter group on both platforms. The lower average amount of discussions by MOOC-only students (1.98 discussions on average) compared to MOOC ∩ KSX students (4.25 MOOC discussions and 6.73 KSX discussions on average) confirms this observation. Besides the number of comments, there were 46 actively rating participants on the KSX platform that casted 333 positive and 44 negative votes on the discussions and comments. The KSX discussions also attracted a considerable amount of passive forum readers.

**Table 3:** MOOC and KSX forum activity.

Participants	MOOC (Total)	MOOC Only	MOOC ∩ KSX	
Platform	MOOC	MOOC	MOOC	KSX
Commenting Users	1,339	1,084	255	66
No. of Discussions	3,225	2,142	1,083	444
No. of Comments	4,502	2,936	1,566	800
Avg. Discussions per User (SD)	2.41 (8.90)	1.98 (5.69)	4.25 (16.58)	6.73 (15.22)
Avg. Comments per User (SD)	3.36 (12.35)	2.71 (8.05)	6.14 (22.75)	12.12 (26.51)

#### Proposal and trading activities

On the KSX platform, 36 students proposed a total of 117 solutions, which refers to 9.75 solutions per challenge on average. In the proposal stage, 100 stocks were approved by the course supervisors. These stocks were additionally supported by 48 uploaded documents and 88 uploaded images (including 50 preview images to provide a more individual character for the stock). The automated market maker processed 2,007 incoming orders from 94 different participants.

## Model and results

### The effect of KSX participation on MOOC completion rates

A main goal of our study is to test whether students’ completion rates can be increased via the interaction and engagement of the KSX platform. The descriptive statistics in Table 1 above already indicated a rather low completion rate of MOOC-only participants (6.4%), which is similar to other MOOC platforms [26]. Moreover, as most participants not just tried to register for the course but completed the registration process and start getting involved by watching videos, these low completion rates cannot simply be dismissed as consequences of a complex registration process. In order to understand the low completion rates in more detail, we take a closer look at the different course stages that students were required to pass.

Table 4 depicts the completion rates for each stage over the entire course period. The dropout rate of more than 80 percent before the very first quiz, indicates that a large fraction of students was not seriously willing to complete the course and receive a certificate. Some of them may only be interested in getting free access to the course material. This can be considered as a type of free-rider problem that many MOOCs deliberately accept in return to improved signup rates. In contrast, we observe 9,660 students who passed at least the first quiz, thereby indicating their serious interest in a successful completion of the course. Quiz by quiz we also detect overall increasing completion rates that can be interpreted as a continuously increasing lock-in effect. For MOOC ∩ KSX students, the completion rates are above 90 percent for every single stage.

**Table 4:** Completion rates by progress stage (based on completing the previous stage).

	Active Accounts	Quiz 1	Quiz 2	Quiz 3	Quiz 4	Quiz 5	Quiz 6	Quiz 7	Final Exam
Total	58,729	9,660	7,809	6,654	5,915	5,424	5,082	4,498	4,324
(%)	100.0%	16.4%	80.8%	85.2%	88.9%	91.9%	93.5%	88.5%	96.1%
MOOC∩KSX	921	866	838	797	734	692	664	613	598
(%)	100.0%	94.0%	96.8%	95.1%	92.1%	94.3%	96.0%	92.3%	97.6%
MOOC Only	57,808	8,794	6,971	5,857	5,181	4,742	4,418	3,885	3,726
(%)	100.0%	15.2%	79.3%	84.0%	88.5%	91.5%	93.2%	87.9%	95.9%

As can be seen in Tables 1 and 4, the completion rate of MOOC-only participants (6.4%) is significantly lower compared to participants of both platforms (64.9%). However, as students joined the KSX platform on a voluntary basis, without random assignment to one of the two groups, it is important to address the possibility of self-selection effects [55]. One way to account for a possible self-selection bias is based on the propensity score matching (PSM) technique [56, 57]. For observational studies, the PSM substitutes a randomization process by balancing a sample of the treatment group with a sample of the control group. Therefore, the PSM estimates the “Average Treatment Effect on the Treated” (ATT) by accounting for a set of covariates that might influence an individual’s likelihood to receive a treatment [58, 59].

#### Propensity score matching

In the following, we apply different matching techniques via PSM that allow us to compare our treatment group (i.e., MOOC ∩ KSX participants) with their counterfactuals chosen from the control group of MOOC-only participants. This is done by predicting students’ decision to self-select into the treatment (i.e., active KSX participation: yes/no). In order to match MOOC ∩ KSX participants with a homogenous group of MOOC-only participants [59, 60], we chose a set of confounding variables that were available for both groups (i.e., registration order, scores of quiz 1, MOOC user ratings, MOOC forum posts, and the usage of late days, see Table C in S1 File) to calculate the propensity scores based on a standard probit model (Table D and Table E in S1 File). This allows us to directly compare students from both groups that have similar propensity scores. Table 5 below shows that the probability to pass all quizzes is significantly higher for the treatment group (i.e., MOOC ∩ KSX participants), independent of the applied matching algorithm (i.e., kernel matching, k-nearest neighbor matching (kNN)).

**Table 5:** Effect of KSX participation on MOOC completion rate.

Matching	Sample	Treated	Controls	Difference	SE	Covariates	T-stat	N
Unmatched	All active	0.666	0.067	0.598	0.009	Full	70.56***	58,729
ATT (Kernel)	All active	0.666	0.205	0.461	0.016	Full	29.48***	58,729
ATT (kNN)	All active	0.664	0.502	0.163	0.024	Full	6.79***	58,729
Unmatched	After Q1	0.708	0.442	0.266	0.018	PreQ1	15.15***	9,660
ATT (Kernel)	After Q1	0.708	0.465	0.243	0.017	PreQ1	14.72***	9,660
ATT (kNN)	After Q1	0.707	0.526	0.181	0.024	PreQ1	7.48***	9,660
Dependent variable: All quizzes passed (Yes/No)				Treatment variable: Active KSX participation (Yes/No)				
Sample (All active):Active MOOC participants				Sample (After Q1): MOOC participants who passed quiz 1				

Treatment covariates (Full): Registration Order, Ratings, MOOC Score Quiz1, #MOOC Forum Posts, Late Days

Treatment covariates (PreQ1): Registration Oder, MOOC Score Quiz 1, #MOOC Forum Posts (prior to Quiz 1)

***, ** and * denote significance at 0.01, 0.05 and 0.1, respectively.

As an additional robustness check, we limited the sample of students to those that passed the first quiz to bypass the selection-effect of the first stage (and as an indication for students’ intention to receive a certificate). In doing so, we limited the set of covariates to those variables that were already observable before students passed the first quiz. The results can be seen in the last three rows of Table 4 (see sample “After Q1”). Although the difference between both groups slightly decreases, there is still a significant distinction that provides clear evidence for the positive effect of KSX participation on students’ motivation to pass all quizzes.

#### Survival analysis

In order to get deeper procedural insights into dropouts, we modelled students’ dropout hazard using a discrete time survival analysis [61, 62]. This method relies on a logistic regression, which predicts the likelihood that students will exit the course within a discrete period of time before writing the final exam. As a preparatory step, each person-specific observation has to be converted into a new person-period data set with multiple observations [62]. For each of the quizzes, a dummy-coded variable is introduced as a time indicator. As can be seen in Table 6, this procedure leads to 103,781 observations, clustered in 58,729 groups. The seven time indicators represent discrete time intervals at which a stage-specific dropout event can occur. In addition, we are using eight time-invariant predictors, four activity-based predictors of the KSX platform and four predictors to understand the effect of MOOC activities: IsStockProposer (KSX), IsDiscussionInitiator (KSX), IsDiscus-sionReplier (KSX), IsStockTrader (KSX), IsDiscussionInitiator (MOOC), IsDiscussionReplier (MOOC), IsVoter (MOOC), and LateDaysUsed (MOOC). We model the subsequent survival rates of individual i in time period j as follows [62]:

$$h_{ij}=\frac{1}{1+e^{-[(\alpha_{1}D_{1ij}+\ldots +\alpha_{J}D_{Jij})+(\beta_{1}Z_{1ij}+\ldots +\beta_{P}Z_{Pij})]}}$$ (1)

where [D_1ij_…D_Jij_] is a sequence of dummy predictors and J refers to the last observed time period. The intercept coefficients [α_1_…α_J_] describe the baseline level of hazard per time period and the slope coefficients [β_1_…β_P_] capture the effects of the model predictors on the hazard function. The results are shown in Table 6. Negative coefficients of the logistic regression indicate a risk-reducing effect on the dependent variable (i.e., course dropout).

**Table 6:** Effect of MOOC and KSX Activity on MOOC dropout.

Discrete Survival Analysis (Logit)		Coefficient	Robust Standard Error
IsStockProposer (KSX)		-0.760*	0.430
IsDiscussionInitiator (KSX)		-1.325***	0.404
IsDiscussionReplier (KSX)		-1.190**	0.470
IsStockTrader (KSX)		-1.539***	0.260
IsDiscussionInitiator (MOOC)		-1.924***	0.064
IsDiscussionReplier (MOOC)		-1.828***	0.154
IsVoter (MOOC)		-0.438***	0.097
LateDaysUsed (MOOC)		0.243***	0.008
Stage 1 (after MOOC Quiz 1)		-2.963***	0.029
Stage 2 (after MOOC Quiz 2)		-3.247***	0.035
Stage 3 (after MOOC Quiz 3)		-3.565***	0.042
Stage 4 (after MOOC Quiz 4)		-3.906***	0.050
Stage 5 (after MOOC Quiz 5)		-4.150***	0.057
Stage 6 (after MOOC Quiz 6)		-3.506***	0.047
Stage 7 (after MOOC Quiz 7)		-4.701***	0.079
Constant		-0.685***	0.081
*N*	103,781	*Pr* > *Χ*^2^	0.0000
No. of clusters	58,729	Pseudo *R*^*2*^	0.4407
*DV*	Dropout (Yes: 1/No: 0)	Wald chi^2^ (15)	32,615

***, ** and * denote significance at 0.01, 0.05 and 0.1, respectively.

The results indicate that active participation on both platforms as well as the usage of late days reduce the risk of course dropout. The usage of binary predictor variables (except for the usage of late days) allows us compare their effect size. Besides the stage-specific time indicators, the most prominent effect for both platforms is related to active forum participation (i.e., initiating discussions and/or replying on existing discussions). Forum activity on the MOOC platform has a stronger effect size than on the KSX platform, as it is directly linked to the course content. With respect to the KSX platform, students that actively engaged in the trading of virtual knowledge stocks strongly increase the course completion likelihood, followed by students who initiated discussions on the KSX forum. Moreover, we observe a successively increasing effect size for the stage-specific indicators, suggesting a platform-specific lock-in effect that increases from quiz to quiz (except for stage 6).

### The effect of KSX participation on MOOC final exam grades

We additionally analyze whether KSX participation can improve students’ final exam grades. The mean of final exam grade–based on 4,498 students that successfully passed the seven obligatory quizzes–is 24.41, with a standard deviation of 6.3 grade points. Three out of four students achieved a final exam grade of at least 23.52 points. A two-sample t-test (t = -3.902, p < 0.01) indicates that the average final exam grades of 3,885 active MOOC-only participants (M = 24.26, SD = 6.43) are significantly lower (4.4%) compared to 613 active MOOC∩KSX participants (M = 25.33, SD = 5.35).

Again, this model-free result does not allow us to rule out the possibility of a self-selection bias. Similar to the analysis on completion rates above, we match the treatment group of MOOC ∩ KSX participants with the control group of MOOC-only participants based on the propensity scores of a probit regression (see Table F in S1 File). Table 7 shows that the differences between both groups are significantly different, regardless of the applied matching algorithm. Contrary to initial concerns that the gamification elements of a KSX might have a distracting effect, potentially resulting in poor grades, we even observe an increase of students’ final exam grades.

**Table 7:** Effect of KSX participation on MOOC final exam results.

Matching	Sample	Treated	Controls	Difference	SE	Covariates	T-stat	N
Unmatched	All passed	25.328	24.261	1.067	0.273	Full	3.90***	4,498
ATT (Kernel)	All passed	25.328	24.570	0.758	0.245	Full	3.09***	4,498
ATT (kNN)	All passed	25.311	24.471	0.839	0.371	Full	2.26***	4,494
Dependent variable: Final exam grade				Treatment variable: Active KSX participation (Yes/No)				
Sample (All passed): Active MOOC participants (that passed all quizzes)								
Treatment covariates (Full): Registration Order, Ratings, MOOC Score Quiz1, #MOOC Forum Posts, Late Days								

***, ** and * denote significance at 0.01, 0.05 and 0.1, respectively.

## Discussion

The results of the field study indicate that the combination of MOOCs and KSX is a promising approach to improve student completion rates as well as exam grades. We note in particular that the active trading of stocks and more intense discussions in KSX and MOOC forums, which encourage student-to-student interaction, are accompanied by an improvement in student completion rates. This outcome indicates that greater platform engagement is positively correlated to student success, findings that are similar to previous research [16]. In addition, our findings support the assumption that a gamified coopetitive design encourages a more interactive learning environment, also in line with the results from prior research [14], which in turn fosters higher completion rates and better exam grades. This coopetitive design is enabled by our proposed method, the KSX, a novel type of VSM mechanism designed to support knowledge management and e-learning.

Conceptually, we make us of elements from gamification research, based on insights from social psychology. Specifically, cognitive evaluation theory [46] that combines feedback and rewards with intrinsic motivation as well as social comparison theory [47] that reflects individuals needs to compare each other. Starting with a set of rudimentary knowledge artifacts, students are able to collaboratively improve proposed solutions in iterative cycles. Forum functionalities allow students to jointly discuss their solutions and to build learning networks. Students thus benefit from an engaging and interactive market-based validation of the knowledge artifacts through the crowd-based intelligence mechanism. Specifically, the learning environment allows students to learn with and from their peers, thereby improving memorization and comprehension in a coopetive atmosphere, without the pressure of an authority figure [16]. This might contribute to students’ improved exam grades. Leaderboards additionally reflect participants’ platforms activities, which might increase students’ ongoing engagement by their underlying need for social comparison.

We also should acknowledge some limitations, which provide avenues for future research. First, the study does not evaluate a fully integrated system of MOOC and KSX on a single web-platform. Instead, participants have to register on both platforms separately. The repeated switching between both platforms may have weakened KSX effectiveness. For MOOC providers, it therefore might be advisable to implement a fully integrated system of MOOC and KSX. Second, because most students perceived the learning environment as equally collaborative and competitive, we are not able to trace the motivating effect back to more specific environmental factors. Instead, interaction appears to be driven by a coopetitive overall impression of the KSX platform. Third, there might be heterogeneity in participants’ responses to the gamification mechanism as different participants might respond differently to the same mechanism, based on different personality traits and preferences. A randomized controlled trial would help to measure the causal impact of different gamification mechanisms via treatments groups, for example by varying the degrees of collaboration and competition, which could provide insights into the mechanisms’ effectiveness regarding learning outcomes. Estimating the resulting heterogeneity of treatment effects by taking participants’ personality traits, based on survey data, into account would deliver additional avenues for a more personalized approach to gamification [63].

## Conclusions

To conclude, this paper contributes in three major ways to the ongoing problem of low engagement and interaction in MOOCs as well as knowledge management in general. First, we demonstrate that the underlying mechanism of Virtual Stock Markets (VSM), which have mainly been used to predict the probability of future events, can also be applied to knowledge sharing by gathering and aggregating widely dispersed information from a large crowd of MOOC participants. This new type of VSM is referred to as Knowledge Stock Exchange (KSX). The KSX offers open access to all MOOC participants to collaboratively develop an open knowledge base in an iterative and incentive-compatible way, allowing participants to reveal their true preferences in the trading process. Second, we can show that the KSX method increases student interaction and engagement in MOOCs. The KSX is engaging in the sense that it allows to actively trade knowledge stocks and to participate in various forum discussions. On average, MOOC and KSX participants comment 2.3 times more often in the MOOC forum compared to MOOC-only participants. From a theoretical perspective, our findings are consistent with engagement enhancing results observed in gamification research [14]. Third, the paper further supports the connection between engagement and better learning outcomes, manifested by higher course completion rates and better exam grades. This is achieved by applying the KSX in combination with competitive (leaderboards, challenges and contribution points) and cooperative (forums) gamification elements. Specifically, the completion rates for MOOC and KSX participants are 10.1 times higher than for MOOC-only participants. In addition, the average exam grade is 4.4 percent higher for MOOC and KSX participants.

The paper not only contributes to the gamification and knowledge management literature by integrating theories from social psychology, such as cognitive evaluation and social comparison theory, but also shows the potential of combining crowd-based intelligence mechanisms–the KSX–with e-learning platforms such as MOOCs as well as gamification elements. Thus, the study provides implications for the design of MOOC platforms going forward and the knowledge management field in general. For example, the integration of KSX and other engagement enhancing methods for the purpose of knowledge sharing within MOOCs seems to be a promising avenue. Future research could investigate more closely the iterative and reciprocal nature of knowledge sharing on KSX and MOOC platforms as well as focus on more personalized forms of gamification.

## Supporting information

S1 File Supporting tables and figures. (DOCX)

S1 Data Data and code. (ZIP)
