# Supplementary material for: How Knowledge Stock Exchanges can increase student success in Massive Open Online Courses
Source: PLoS One. 2019 Sep 26;14(9):e0223064. doi: 10.1371/journal.pone.0223064 (PMC6762193; doi:10.1371/journal.pone.0223064)
Supplement: S1 File — (DOCX) [file pone.0223064.s001.docx]

# Supporting Information

**Table A.** **Contribution Rules.**

| **Rule Description** | **Points** |
| --- | --- |
| A user receives five contribution points for proposing a new knowledge stock. | +5 |
| As soon as a virtual knowledge stock gets approved, the proposer will receive five additional contribution points. | +5 |
| If a proposed knowledge stock gets rejected (due to offensive content, a double-post or other reasons) by a market supervisor, all contribution points that the user received for proposing the stock will be retracted. | -5 |
| Starting a knowledge improvement discussion will be rewarded with three contribution points. | +3 |
| Users receive two additional contribution points if their discussion counts five replies of different users. All in all they can earn up to five contribution points for starting a lively knowledge improvement discussion. | +2 |
| Comments within a knowledge improvement discussion will be rewarded as soon as they receive their third thumbs up! The comment's author will receive two contribution points. | +2 |

The contribution rules are based on the gamification mechanics that describe how and when points are rewarded to participants.

**Table B.** **Knowledge Challenges of the MOOC.**

| **Title** | **Task Description – Abstract** | **Submitted  Solutions** |
| --- | --- | --- |
| Get Started | Prepare a terminology dictionary for Advanced Competitive Strategy! | 23 |
| Module 1:  Switching Costs | Prepare an exercise for your fellow students that embeds some of the concepts of Module 1 (e.g., customer loyalty, customer value, switching costs)! | 13 |
| Module 2:  Price Discrimination | Prepare a real life case study that features different applications of price discrimination! | 10 |
| Module 3:  Competition Policy | Write a case discussion of a prominent violator of Competition Policy and/or Antitrust Law and prepare questions for your fellow students! | 2 |
| Module 4:  Business Restructuring | Restructure a business of your choice (real or fictional) in terms of a principle that you have learned in Module 4! | 4 |
| Module 5:  Network Products | Design your own network product and write up a detailed, ingenious strategy taking into account the specifics of network markets! | 4 |
| Module 6:  Mergers & Acquisitions | Write a case about a significant merger or acquisition of your choice! | 4 |
| Module 7: Local  Conventions & Growth | Describe a local or national firm (that only operates in your home country) and make it a global player! | 8 |
| Case Discussions | Propose a case discussion that addresses a specific topic of Advanced Competitive Strategy! | 9 |
| Case Studies | Prepare a case study that addresses a topic of Advanced Competitive Strategy and prepare questions for your fellow students! | 19 |
| Business Plans | Propose a business plan that addresses one or more topics of Advanced Competitive Strategy! | 7 |
| Other | You want to share your knowledge but you didn't find a suitable challenge? Propose all kind of helpful knowledge, including but not limited to: student transcripts, student exercises, terminology dictionaries, literature overviews, numerical examples, or brief explanations. | 14 |

**Table C.** **Variable Names.**

| **Variable** | **Description** | **Type [Range]** |
| --- | --- | --- |
| ActiveKSXParticipation | Users who register and actively participate on the KSX platform. | *binary* |
| FinalExamGrade | A user's final exam grade on the MOOC platform, ranging from 0 to 30. | *count [0;30]* |
| IsStockProposer  (KSX) | Users who submit at least on solution (i.e. stock proposal) on the KSX platform. | *binary* |
| IsDiscussionInitiator (KSX) | Users who initiate at least one forum discussion on the KSX platform. | *binary* |
| IsDiscussionReplier (KSX) | Users who comment on at least one forum discussion on the KSX platform. | *binary* |
| IsStockTrader (KSX) | Users who buy or sell at least one stock share on the KSX platform. | *binary* |
| IsDiscussionInitiator (MOOC) | Users who initiate at least one forum discussion on the MOOC platform. | *binary* |
| IsDiscussionReplier (MOOC) | Users who comment on at least one forum discussion on the MOOC platform. | *binary* |
| IsVoter (MOOC) | User who vote on at least one forum comment on the MOOC platform. | *binary* |
| LateDaysUsed (MOOC) | Each participant can use up to 10 late days to remove the penalty applied between the quizzes' due date and the hard deadline. | *count [0;10]* |
| MOOC Registration Order | Order in which students registered for the course on the MOOC platform. | *count* |
| MOOC Num Ratings | Number of forum votes a user submitted within the course on the MOOC platform. | *count* |
| MOOC Score [Quiz] | A user's quiz score - ranging from 0 to 10 - for a learning module of the course on the MOOC platform. | *count [0;10]* |
| MOOC #Comments  on [Forum] | Number of comments in a forum of the course on the MOOC platform. | *count* |

KSX: “Knowledge Stock Exchange”, MOOC: “Massive Open Online Course.”

**Table D.** **Propensity Score Estimation: Full / Active Participants.**

| **Independent Variables** | **Coefficient** | **Standard Error** |
| --- | --- | --- |
| MOOC Registration Order | -2.99e^-06^*** | 0.000 |
| MOOC Num Ratings | 0.011 | 0.008 |
| MOOC Score Quiz 1 | 0.179*** | 0.004 |
| MOOC #Comments on General | 0.142*** | 0.025 |
| MOOC #Comments on Syllabus | -0.368** | 0.182 |
| MOOC #Comments on Module 1 | -0.072 | 0.096 |
| MOOC #Comments on Module 2 | 0.172* | 0.102 |
| MOOC #Comments on Module 3 | 0.120 | 0.118 |
| MOOC #Comments on Module 4 | 0.064 | 0.196 |
| MOOC #Comments on Module 5 | 0.161 | 0.153 |
| MOOC #Comments on Module 6 | 0.400 | 0.435 |
| MOOC #Comments on Module 7 | -0.222** | 0.086 |
| MOOC LateDaysUsed | 0.010 | 0.008 |
| Constant | -2.787*** | 0.092 |
| Observations (*N*) | 58,729 | |
| *DV* | Active KSX Participation (Yes: 1/No: 0) | |
| *Pr* > *Χ*^2^ | 0.000 (LR *Χ*^2^ = 2,967.11) | |

***, ** and * denote significance at 0.01, 0.05 and 0.1, respectively.

**Table E.** **Propensity Score Estimation: PreQ1 / Before Quiz 1.**

| **Independent Variables** | **Coefficient** | **Standard Error** |
| --- | --- | --- |
| MOOC Registration Order | -4.68e^-06^*** | 0.000 |
| MOOC Score Quiz 1 | 0.103*** | 0.011 |
| MOOC #Comments on General | 0.139*** | 0.022 |
| MOOC #Comments on Syllabus | -0.238 | 0.170 |
| MOOC #Comments on Module 1 | -0.026 | 0.087 |
| Constant | -2.133*** | 0.106 |
| Observations (*N*) | 9,660 | |
| *DV* | Active KSX Participation (Yes: 1/No: 0) | |
| *Pr* > *Χ*^2^ | 0.000 (LR *Χ*^2^ = 208.78) | |

***, ** and * denote significance at 0.01, 0.05 and 0.1, respectively.

**Table F.** **Propensity Score Estimation: Full / Passed All Quizzes.**

| **Independent Variables** | **Coefficient** | **Standard Error** |
| --- | --- | --- |
| MOOC Registration Order | -4.79e^-06^*** | 0.000 |
| MOOC Num Ratings | 0.026** | 0.011 |
| MOOC Score Quiz 1 | 0.100*** | 0.020 |
| MOOC #Comments on General | 0.153*** | 0.030 |
| MOOC #Comments on Syllabus | -0.813*** | 0.233 |
| MOOC #Comments on Module 1 | 0.008 | 0.103 |
| MOOC #Comments on Module 2 | 0.073* | 0.127 |
| MOOC #Comments on Module 3 | -0.051 | 0.186 |
| MOOC #Comments on Module 4 | 0.336 | 0.273 |
| MOOC #Comments on Module 5 | 0.054 | 0.156 |
| MOOC #Comments on Module 6 | 0.491 | 0.475 |
| MOOC #Comments on Module 7 | -0.203** | 0.091 |
| MOOC LateDaysUsed | 0.006 | 0.009 |
| Constant | -1.879*** | 0.209 |
| Observations (*N*) | 4,498 | |
| *DV* | Active KSX Participation (Yes: 1/No: 0) | |
| *Pr* > *Χ*^2^ | 0.000 (LR *Χ*^2^ = 110.26) | |

***, ** and * denote significance at 0.01, 0.05 and 0.1, respectively.

**Fig A. Post-Survey: Participation by Age.**

**Fig B.** **Post-Survey: Participation by Highest Degree of Education.**
